# Supplementary material for: Complexome profiling of the Chlamydomonas psb28 mutant reveals TEF5 as an early PSII assembly factor
Source: Plant Cell. 2025 Mar 17;37(6):koaf055. doi: 10.1093/plcell/koaf055 (PMC12164590; doi:10.1093/plcell/koaf055)
Supplement: koaf055_Supplementary_Data [file koaf055_supplementary_data.zip › psb28_interactive.zip!/plots/intensity/qualityPlots/peptides/ERD2A_Cre01.g051400.t1.2.html]

### Cre01.g051400.t1.2

Trivial name: ERD2A  
SeqCoverage [median]: 2.80  
Mass: 25.80 kDa  
  
 

| Sequence | MissedCleavage | Mass [Da] | wtIntensity | muIntensity | Score |
| --- | --- | --- | --- | --- | --- |
| MMNIFR | 0 | 810.39 | 543.29 | 1467.86 | 88.63 |
|  |  |  | 543.3 | 1467.9 |  |
